# Improving Nutrient Efficiency as a Strategy to Reduce Nutrient Surpluses on Dairy Farms

**DOI:** 10.1100/tsw.2001.266

**Published:** 2001-10-24

**Authors:** C.J.M. Ondersteijn, A.G.J.M. Oude Lansink, G.W.J. Giesen, R.B.M. Huirne

**Affiliations:** Farm Management Group, Department of Social Sciences, Wageningen University, The Netherlands

**Keywords:** nutrient efficiency, Data Envelopment Analysis, nitrogen surplus, phosphate surplus, dairy farming, agricultural nutrient policy, the Netherlands

## Abstract

Dutch nutrient policy aims at reducing leaching of agricultural nutrients by internalizing the negative externalities associated with inefficient nutrient use. This is done by taxation of nitrogen and phosphate surpluses that exceed a hectare-based threshold of maximum-allowed surpluses. One management strategy farmers may use to reduce the nutrient surpluses on their farms is to improve the nutrient efficiency of the agricultural production process. This study employs Data Envelopment Analysis (DEA) to calculate nitrogen and phosphate efficiencies and an overall nutrient efficiency measure for a 3-year panel of 114 Dutch dairy farms. Subsequent analyses show the impact of both farm intensity and nutrient efficiency on the nitrogen and phosphate surpluses. It appears that farm intensity has a positive effect on efficiency, but efficiency and intensity exert opposite influences on nutrient surpluses. This is especially the case for nitrogen. The magnitude of a possible reduction of nitrogen surpluses through a strategy of efficiency improvement is therefore limited by the intensity of the farming system, unless the technology with which nutrients are used by the farming system can be further improved or input/output ratios will be altered.

## INTRODUCTION

Recent developments in European, and in particular Dutch, nutrient policy have forced farmers to consider nutrient management as an essential part of their decision-making process[1]. Dutch farmers have to operate within the Mineral Accounting System (MINAS), a nutrient accounting system that taxes farmers with nitrogen and phosphate surpluses exceeding a predetermined surplus standard. In this way, the Dutch government aims at internalizing the negative externalities associated with inefficient nutrient use. The main objective of MINAS is to reduce nutrient pollution of groundwater, which is the most important source of drinking water in the Netherlands. The system is output oriented, meaning that farmers are taxed according to their final nutrient results irrespective of the way they arrive at it. This development forces farmers to incorporate nutrient management into their total management, since financial repercussions can be severe[2]. One way to improve nutrient management, and thus reduce the surpluses which accompany the agricultural production process, is to improve the efficiency with which nutrients are used.

To improve nutrient efficiency, and environmental performance in general, it is crucial to learn from successful colleagues that can act as benchmarks, rather than focussing on central tendencies. The ultimate goal of (environmental) performance research is, after all, to improve this performance[3]. This is why a frontier approach, in which farm efficiency is measured as the deviation from best performance, may provide more insight into performance and can indicate ways to improve performance better than methods like regression models, which focus on average performance.

The purpose of this paper is to provide insight into the possibilities for Dutch dairy farmers to improve nutrient performance, through nutrient efficiency improvement, in order to meet the environmental policy objectives that were recently introduced in the Netherlands. Data Envelopment Analysis (DEA) is used to estimate nutrient efficiency. The magnitude of the current possibilities for reduction of nutrient surpluses is calculated and, finally, nutrient efficiencies are related to farm intensity and nutrient surpluses using regression analysis.

## MATERIALS AND METHODS

### Nutrient Efficiency

Debreu[4] and Farrell[5] specified an operational definition of efficiency that complies with Koopmans theoretical definition of technical efficiency[6]. This definition states that a producer is technically efficient if an input reduction requires an increase in another input or a decrease in at least one output[6]. The Debreu-Farrell (DF) measure of technical efficiency is defined as one minus the maximum equiproportionate reduction in all inputs that still allows continued production of given outputs. A score of unity indicates full efficiency, whereas a score below one suggests that a reduction in inputs is possible without sacrificing output.

Efficiency measures can be determined by parametric as well as nonparametric approaches. Efficiency measurement in agriculture is complicated by the fact that agricultural processes are more or less stochastic and, if not accounted for, this stochasticity can have a serious impact on estimated efficiency. The choice for parametric or stochastic frontier analysis (SFA) seems therefore obvious. Coelli et al. recommend the use of SFA over nonparametric approaches in agricultural research[7]. However, the problem with SFA is that it assumes a functional form for production technology, which can confound the efficiency results[8]. Nonparametric programming approaches, currently known as DEA, have an enormous flexibility in that they avoid a parametric specification of technology and the distribution of efficiency. Further information on the benefits of DEA over SFA or other parametric approaches can be found in the literature[9,10,11].

DEA yields a measure of DF efficiency of a farm relative to the best practice farms in the sample. In an input-oriented DEA model, the objective is to produce the observed output with as little input as possible. This is a reasonable assumption in European milk production since it is limited by a production quota for every individual farm. In a homogenous market in which prices change very little in a short time period, the most common way to maximize profit is therefore input minimization.

The production of milk on Dutch dairy farms generates negative externalities in the form of nutrient surpluses. They can be modeled as a weakly disposable output in a distance-function approach[12,13], or as a (weakly) disposable input to be minimized[14,15]. In this study, the latter method is employed because the nutrient surpluses can be seen as net inputs rather than outputs resulting from the production process. Using the concept of “non-radial” efficiency, which calculates the efficiency of a subvector of a particular input (in this case, nutrient surpluses), an efficiency estimate for the nutrient surpluses can be calculated to get an indication of nutrient performance of the farm[16]. The nutrient efficiency is defined as the ratio of minimum feasible to observed nutrient surpluses, conditional on observed levels of output and conventional input[17]. This can be formulated as a linear programming problem:

$$\begin{aligned} &\underset{\gamma,\lambda}{\boldsymbol{Min}}\ \boldsymbol{\gamma} \\ &\textit{Subject to:} \quad \boldsymbol{y_i} \le \boldsymbol{Y}\lambda \\ &\qquad \boldsymbol{x_i} \ge \boldsymbol{X}\lambda \\ &\qquad \lambda \boldsymbol{w_i} = \boldsymbol{W}\lambda \\ &\qquad N'\lambda = 1 \\ &\qquad \lambda \ge 0 \end{aligned} \tag{1}$$

Nutrient efficiency for the $i$th firm is represented by $\gamma$ ($\gamma \in [0,1]$) for the $i$th firm. Y is the observed vector of output, X is the observed vector of conventional input, and W is the observed vector of the environmentally detrimental input (i.e., the nitrogen and phosphate surplus). Firm weights are represented by an $N \times 1$ vector $\lambda$, where N is the number of farms in the sample. The first and second constraints reflect strong disposability of outputs and conventional input, respectively. The equality in the third constraint imposes weak disposability of the nutrient surpluses. The fourth constraint allows for a technology characterized by variable returns to scale, which envelopes the data most tightly, compared to constant returns to scale and nonincreasing returns to scale. The nutrient efficiency model involves finding the frontier that minimizes both the nitrogen and phosphate surplus simultaneously.

Like Reinhard et al., separate production sets and efficiencies are calculated for each year of the panel[17]. Window analysis, which incorporates every preceding year in the calculation of efficiencies in subsequent years, would mix up technological change and (stochastic) year effects in the calculated efficiencies[18]. Since our sample is reasonably large, this can be avoided by calculating efficiencies for every year in the sample separately, and then averaging over the total period.

Fig. 1 gives a graphic explanation of the concept of nonradial input-orientated DEA efficiency using two inputs, $x_1$ and $x_2$. P, Q, and R represent farms that use a different combination of $x_1$ and $x_2$. DEA creates a piecewise linear isoquant from the observations, representing the most efficient technologies used by the farms in the sample (in this case, farms Q and R, which use the least input while producing the same amount of output as all other farms). Nutrient efficiency can be calculated as a nonradial measure (as opposed to the radial measure OP′/OP, which represents total technical efficiency). Suppose $x_1$ represents the nutrient surpluses. Nutrient efficiency can then be calculated as O′P″/O′P and indicates that farm P could reduce the use of $x_1$ with a proportion of 1 – O′P″/O′P.

### Data Description

Data of 114 specialized dairy farms[1] were collected over a period of 3 years (1997 to 1999) as part of a large government supported project called Farm Data in Practice (FDP, Project Praktijkcijfers). The goal of the project was to gain empirical

[1] Farms are classified as a specialized dairy farm if at least 95% of all Dutch Size Units [DSUs] can be attributed to dairy production; the DSU is an economic size unit, based on standard gross margins[19].

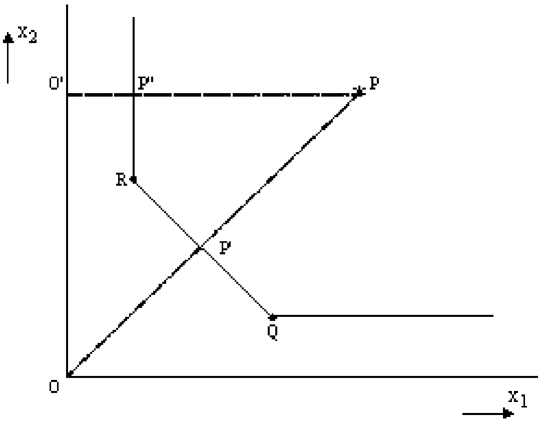

**FIGURE 1.** Input-oriented DEA efficiency.

insight into nutrient management on "real-life" farms, and to support and improve nutrient management. Farms were selected to give an accurate representation of dairy farms in the Netherlands, but the management support they received affects the representativeness of the farms. However, because the goal of this study is to show the possibilities for an improvement of nutrient management, this does not affect the validity of the results. Data collection was organized by the project organization and was carried out by the bookkeepers of the farmers. Both financial and nutrient accounting data were collected as well as data on farm layout and management characteristics. Screening and rectification of data was done twice, once by the project organization and once by the authors. Table 1 gives an overview of the input and output used in the DEA analysis using three-year averages.

The outputs distinguished are total milk production corrected for fat and protein content, and livestock sales and value change expressed in 1997 •. They represent the major outputs for such highly specialized farms. The inputs used are cultivated land in hectares, total stock aggregated in livestock units (LU), total labor used (both unpaid and paid) in full-time equivalents (FTE), total nitrogen from chemical fertilizer in kilograms, and total phosphate from chemical fertilizer in kilograms. Net feed purchases contains both concentrates and roughage, aggregated using gross energy (GE) content, and corrected for sales and stock changes[2]. Environmental detrimental inputs are the nitrogen and phosphate

**TABLE 1**
**3-Year Averages and Standard Deviation of Total Inputs and Total Outputs***

| | Mean | Standard Deviation |
|---|---|---|
| **Input** | | |
| Cultivated land (ha) | 41.65 | 11.93 |
| Stock (LU) | 92.43 | 28.83 |
| Labor (FTE) | 1.76 | 0.54 |
| Nitrogen fertilizer (kg N) | 11013 | 5304 |
| Phosphate fertilizer (kg $P_2O_5$) | 1677 | 1307 |
| Net feed purchases (GE) | 178794 | 101437 |
| Nitrogen surplus (kg N) | 11163 | 4467 |
| Phosphate surplus (kg $P_2O_5$) | 2152 | 1238.36 |
| **Output** | | |
| Milk production (kg fat and protein corrected milk) | 590437 | 201928 |
| Livestock sales and value change (1997 •) | 21196 | 8568 |

* Livestock sales and value change = livestock sales livestock purchases + change in value of livestock inventory.

[2] Data on machinery and equipment were considered to be of insufficient quality to be used in the analysis; the misspecification of the DEA model due to this omission will negatively affect efficiency.

surpluses, calculated as the difference between the use and production of nutrients (the difference between input and output corrected for stock changes). All inputs are considered strongly disposable except for the nutrient surpluses. Even though land may be seen as a factor that is hard to acquire, disposing of it is easy. In an input-orientated DEA, the goal is to minimize input, which means that the availability property is not important, but the disposability property is. Land is therefore modeled as strongly disposable. A similar observation can be made for labor. Both the nitrogen and phosphate surplus are assumed to be weakly disposable, since there is a cost involved with disposing of it.

## RESULTS AND DISCUSSION

### Nutrient Efficiency

Nutrient efficiency was calculated according to the model explained above, and incorporates both nitrogen and phosphate. Average nutrient efficiencies (NE) for every separate year and a 3-year average are presented in Table 2. The nutrient efficiencies for the separate years are approximately 80%.

The number of fully efficient farms differs slightly among years, with 1998 having the highest number (61.4%) of fully efficient farms. The mean nutrient efficiency for the nonefficient farms increases over time, indicating that the nonefficient farms have made improvements. The 3-year average is 80.16%, meaning that a simultaneous reduction of almost 20% is possible for the nitrogen and phosphate surplus. Only 28.9% of the farms in the sample were fully efficient over the 3-year period; the farms that were not could lower their surpluses by about 28% if they would produce on the frontier (Table 2).

The DEA nutrient efficiencies found in the present study are high compared to other studies[14]. The fact that the results were obtained from a highly homogenous group of farms, having at least 95% of all their activities in dairy farming, and input was only aggregated in physical units and corrected for quality differences, will result in high efficiencies. The advantage of this approach is that variance found can be more correctly attributed to management differences, instead of to noise in the data.

Table 3 shows the 3-year averages of the absolute reductions in nutrient surpluses that are possible under the technology that was used from 1997 to 1999. The actually achieved surpluses (268 kg N/ha and 52 kg $P_2O_5$/ha) could be reduced by 52 kg N/ha and 13 kg $P_2O_5$/ha, respectively, if all farms were to produce on the efficient frontier. The final (to be introduced in 2003) Levy Free Surplus (LFS) for nitrogen is set at 180 kg N/ha for grassland and 100 kg N/ha for arable land. For phosphate, the final LFS is set at 20 kg $P_2O_5$/ha. The last row of Table 3 shows the 3-year averages of the final LFS. It is obvious that, for both nitrogen and phosphate, an efficiency improvement towards the current level of best performance will not suffice to comply with the final LFS. The final LFS for nitrogen would still be exceeded by 47 kg N/ha, and the final LFS for phosphate by 19 kg $P_2O_5$/ha. Therefore, in order to be able to meet the final LFS in 2003, not only an efficiency improvement, but also an improvement in technology used (i.e., a shift in the DEA-frontier) is needed.

Nutrient efficiency can be further decomposed into nitrogen and phosphate efficiency using a nonradial DEA approach similar to the calculation of total nutrient efficiency. The results are shown in Table 4. Nitrogen efficiency appears to be higher than nutrient efficiency (the 3-year averages are 89.42 and 80.16, respectively), whereas phosphate efficiency is lower (77.51 vs. 80.16). The consequence of this is that if farmers were to focus entirely on nitrogen, disregarding phosphate management, nitrogen surpluses could not be reduced as much as shown in Table 3. On the other hand, if farmers were to focus on phosphate management, phosphate surpluses could be reduced more than indi-

**TABLE 2**
**Nutrient Efficiency Scores for Separate Years and 3-Year Averages**

| | 1997 | 1998 | 1999 | 1997–1999 |
|---|---|---|---|---|
| Mean all farms | 78.04 | 81.96 | 80.49 | 80.16 |
| % of fully efficient farms | 55.3 | 61.4 | 56.1 | 28.9 |
| Mean nonefficient farms | 50.90 | 53.25 | 55.52 | 72.08 |

**TABLE 3**
**3-Year Averages of Reduction Possibilities Through Efficiency Improvement for Nitrogen and Phosphate**

| | Nitrogen kg N/ha | Phosphate kg $P_2O_5$/ha |
|---|---|---|
| Real surplus | 268 | 52 |
| Reduction through efficiency improvement | 52 | 13 |
| Surplus if all farms were fully efficient | 216 | 39 |
| 2003 levy free surplus | 169 | 20 |

**TABLE 4**
**Nitrogen and Phosphate Efficiency for Separate Years and 3-Year Averages**

| | 1997 | 1998 | 1999 | 1997–1999 |
|---|---|---|---|---|
| Nitrogen efficiency | | | | |
| Mean all farms | 90.78 | 88.84 | 88.64 | 89.42 |
| % of fully efficient farms | 70.2 | 65.8 | 64.0 | 42.1 |
| Mean nonefficient farms | 69.11 | 67.38 | 68.41 | 81.73 |
| Phosphate efficiency | | | | |
| Mean all farms | 76.3 | 76.11 | 80.16 | 77.51 |
| % of fully efficient farms | 60.5 | 62.3 | 61.4 | 35.1 |
| Mean nonefficient farms | 39.87 | 36.65 | 48.59 | 65.35 |

cated by Table 3. Clearly, nutrient management that considers both nitrogen and phosphate is more effective in reducing nutrient surpluses. Dutch agricultural policy before MINAS used to focus on regulating phosphates to control pollution. Apparently, this was not a big issue for dairy farmers. Their nitrogen management is more efficient than their phosphate management, even though phosphates are easier to control.

## Nutrient Efficiency, Farm Intensity, and Nutrient Surpluses

Fully nutrient-efficient farms have a significantly higher intensity of the farming system in terms of fat- and protein-corrected milk per hectare (fpcm/ha) than the inefficient farms. The inefficient farms have a 3-year average intensity of 13,444 kg fpcm/ha, compared to 15,147 kg of fpcm/ha for the fully efficient farms ($t = -4.64$, $p < 0.001$). Full efficiency results in relatively low nutrient surpluses per kilogram of produced milk for the highly intensive farms. However, due to the intensity of the farming system, fully efficient farms tend to have higher absolute levels of nutrient surpluses per hectare, especially if they do not dispose of excess manure. A highly intensive farming system involves a large amount of external inputs compared to more extensive systems, which are more self-sufficient, due to a larger proportion of roughage production relative to milk production. Roughage production, however, is less nutrient-efficient than milk production. Nutrients not used for milk production will be excreted in manure, which will then be used on available farmland as fertilizers. Roughage production is an open system in which losses like volatilization and leaching occur. The shift of the less nutrient-efficient roughage production process onto other farms partially explains why more-intensive farming systems are more efficient. Improving nutrient efficiency (lower surplus per unit of output) by increasing intensity will, therefore, simultaneously lead to higher nutrient surpluses per hectare, which are the bases for taxation in MINAS.

A linear regression analysis was done to assess the magnitude of the effect of nutrient efficiency and farm intensity on the nitrogen and phosphate surplus. The analysis was done for the total sample, as a whole, as well as for the inefficient and efficient farms separately (since there is no variation in nutrient effi-

**TABLE 5**
**OLS Regression Results of Nutrient Efficiency and Farm Intensity on Surpluses***

| | Total Sample | | Inefficient Farms | | Efficient Farms | |
|---|---|---|---|---|---|---|
| | b | t | b | t | b | t |
| **Nitrogen surplus (kg N/ha)** | | | | | | |
| Constant | | 12.25 | | 7.27 | | 6.12 |
| Nutrient efficiency | –0.36 | –7.72 | –0.51 | –8.44 | | |
| FPCM/ha | 0.52 | 11.39 | 0.57 | 9.39 | 0.51 | 8.26 |
| $R^2$ | 32% | | 49% | | 26% | |
| **Phosphate surplus (kg $P_2O_5$/ha)** | | | | | | |
| Constant | | 7.45 | | 5.70 | | 2.86 |
| Nutrient efficiency | –0.26 | –4.91 | –0.37 | –4.73 | | |
| FPCM/ha | 0.19 | 3.52 | 0.16 | 2.07 | 0.19 | 2.72 |
| $R^2$ | 8% | | 15% | | 4% | |

* $P < 0.01$ for all t.

ciency for the latter group of farms, only farm intensity will be used as a predictor). Data from 3 years were used to estimate the regression models. The results are shown in Table 5.

In explaining the nitrogen surplus, nutrient efficiency shows a lower relative importance than farm intensity, both for the total sample and for the inefficient farms. This indicates that, at the current level of performance, improvement of efficiency cannot overcome the higher nitrogen surpluses associated with more-intensive farming systems. For the total sample, almost a third of the nitrogen surplus can be explained by differences in nutrient efficiency and farm intensity. For the inefficient farms, this is as much as 49%. For them, improvement of efficiency is a relatively important issue. The regression analysis of farm intensity on nitrogen surplus explains 26% of the nitrogen surplus. Apparently, for the fully efficient farms, other important determinants exist for explaining the nitrogen surplus.

In the case of phosphate surpluses, nutrient efficiency is a relatively more important determinant in explaining the variation in phosphate surpluses than farm intensity. Even though the models have significant F-ratios (not shown in Table 5), the explanatory power is low (4 to 15%), indicating that the phosphate surplus is more dependent on other factors than nutrient efficiency and farm intensity.

## CONCLUSION

The nutrient efficiencies found in this study imply that the farms in the sample are highly nutrient-efficient producers. This indicates that relative spilling of nutrients per unit of production is low. This is more the case for nitrogen than it is for phosphate, the latter being the nutrient Dutch policy used to focus on before MINAS was introduced. It must be noted that the measures calculated here are relative to the best management practices in the sample. Introduction of farms in the sample that are able to produce the same amount of output with lower nutrient surpluses will cause nutrient efficiencies of the current sample to drop. Furthermore, full efficiency does not mean that there is no improvement possible. Farms that produce on the frontier can improve nutrient management by improving technology, and therefore shift the production frontier towards lower input/output ratios.

A note on the level of nutrient surpluses relative to the final surplus standards in MINAS must be made. The final surplus standards were not mandatory in the 1997 to 1999 time period. MINAS was introduced in 1998, and the maximum-allowed surpluses at that time were higher than the final surplus standards. Due to this regulation, farm management was not geared to meet the final surplus standards of 2003. Furthermore, the introduction of MINAS was done using a stepwise procedure. First, farms with a livestock density exceeding 2.5 LU/ha were subjected to the system. Only in 2000 were all other farms enrolled. Therefore, nutrient management was, for many of the farms in the sample, more experimental than goal-oriented.

At the current level of best performance in the sample, improvement of nutrient efficiency alone will not suffice to meet the final LFS of 2003. On the other hand, since the farmers in the sample did not truly put forth the effort to meet the final LFS, the limits of the possibilities for efficiency improvement as a strategy to reduce nutrient surpluses have not been fully explored. If farmers have the intention to meet the MINAS surplus standards while maintaining the intensity of the farming system, technological improvements will have to be made. Another option is to alter the nutrient input/output ratio through, for instance, manure disposal. However, the possibilities for this type of solution are limited in the Netherlands. New policy measures, scheduled to be implemented in 2002, will force farmers to meet application standards for manure. This will entail restrictions on the maximum allowed N-load from manure per hectare. The application standards follow the restrictions on manure application in the Nitrate Directive closely. When implemented, a manure surplus will arise due to a lack of sufficient agricultural area to place all excess manure from farms with high intensity. As a result, a transition towards less intensive farming systems in terms of cattle units per hectare will very likely follow.

Further research should focus on the costs of complying with MINAS. Costs of meeting the final surplus standards depend on farm circumstances and previous nutrient management behavior. The extent to which they can make use of innovation offsets will determine the possibilities for compensating for the extra costs incurred. Therefore, some farms will have to make large investments. Others will reduce costs because of efficiency improvements. Looking at normative studies on the topic[20,21], it seems that costs of compliance may be compensated for by improved efficiency. This depends on farm intensity, however. Intensive farms will be more affected financially by environmental legislation than will more extensive farms, because the optimal animal density of farms decreases with the introduction of environmental legislation[21].

| | | |
|---|---|---|
| Received: | July | 27, 2001 |
| Revised: | September | 20, 2001 |
| Accepted: | October | 3, 2001 |
| Published: | October | 24, 2001 |
